# Unraveling the Genetics of Human Obesity

**DOI:** 10.1371/journal.pgen.0020188

**Published:** 2006-12-29

**Authors:** David M Mutch, Karine Clément

**Affiliations:** University College London, United Kingdom

## Abstract

The use of modern molecular biology tools in deciphering the perturbed biochemistry and physiology underlying the obese state has proven invaluable. Identifying the hypothalamic leptin/melanocortin pathway as critical in many cases of monogenic obesity has permitted targeted, hypothesis-driven experiments to be performed, and has implicated new candidates as causative for previously uncharacterized clinical cases of obesity. Meanwhile, the effects of mutations in the melanocortin-4 receptor gene, for which the obese phenotype varies in the degree of severity among individuals, are now thought to be influenced by one's environmental surroundings. Molecular approaches have revealed that syndromes (Prader-Willi and Bardet-Biedl) previously assumed to be controlled by a single gene are, conversely, regulated by multiple elements. Finally, the application of comprehensive profiling technologies coupled with creative statistical analyses has revealed that interactions between genetic and environmental factors are responsible for the common obesity currently challenging many Westernized societies. As such, an improved understanding of the different “types” of obesity not only permits the development of potential therapies, but also proposes novel and often unexpected directions in deciphering the dysfunctional state of obesity.

## Introduction

According to the World Health Organization (http://www.who.int), there are an estimated 1 billion adults who are overweight (body mass index > 25 kg/m^2^), and 300 million of these are considered clinically obese (body mass index > 30 kg/m^2^). Such staggering statistics clearly suggest that, despite the overt recognition of the taxing effects of obesity on both medical and social programs, Westernized societies are still succumbing to this global epidemic. While technological progress made over the last 20 years has yielded the tools necessary to comprehensively explore the perturbed biochemistry underlying the obese state, it has also demonstrated that interactions between genetic makeup and environment (G×E) are critical for the regulation of adipose mass function. As such, medical and nutritional recommendations based on genetically undefined and/or environmentally heterogeneous population-based studies have, not unsurprisingly, had minimal success in treating common diseases. It is precisely this lack of success that is paving the way for the widely discussed concepts of personalized medicine [1,2] and nutrition [3–5]. However, prior to our society realizing either of these ambitious concepts, the genetic components underlying common diseases such as obesity must be elucidated with confidence.

Over the past ten years, the study of genetically complex diseases has benefited greatly from the extraordinary advances made in molecular biology. While obesity was first thought to be a disease that obeys the rules of Mendelian inheritance, new technologies paint a far more complicated picture of this metabolic disease and have led to unsuspected and fascinating new developments. Obesity stemming from a single, naturally occurring dysfunctional gene (i.e., monogenic obesity) is both severe and rare when compared to the more common form of obesity, in which numerous genes make minor contributions in determining phenotype (i.e., polygenic obesities). Although some genetic candidates underlying monogenic obesities in the mouse have been defined, transferring this knowledge to humans has led to more questions than answers. Indeed, the molecular approach has revealed novel candidate genes for the various types of human obesity, has suggested that several clinical cases previously defined as monogenic obesity are genetically more complex than previously thought, and has clearly positioned G×E interactions as fundamentally important to understanding the mechanisms involved in fat-mass expansion. As discussed in the present review, despite the fact that our understanding of the genetic, biological, and biochemical factors underlying obesity is currently incomplete, the use of novel molecular approaches is rapidly unraveling this complex metabolic disease.

## Monogenic Obesity

To date, nearly 200 cases of human obesity have been associated with a single gene mutation. Furthermore, these mutations all lie in one of 11 genes [6,7]. These cases, which obey Mendelian genetics, are characterized by extremely severe phenotypes that present themselves in childhood and are often associated with additional behavioral, developmental, and endocrine disorders [8]. Initial knowledge concerning monogenic diseases was derived from large-scale linkage analyses in mice that had naturally occurring mutations that led to extreme adiposity. These analyses resulted in the detection of disease loci and the identification of candidate genes [9]. Using such an approach, the majority of mutations in genes underlying monogenic murine obesity have now been cloned [10].

**Figure 1:**
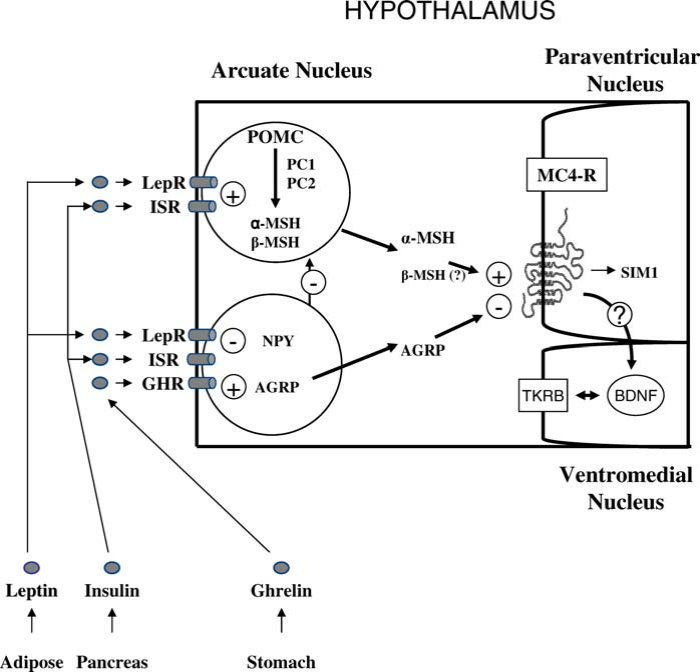
The Leptin/Melanocortin Pathway The integration of signals from peripheral tissues in the hypothalamus is fundamental to the regulation of energy homeostasis. Distinct neuronal populations propagate the signaling of various molecules to control food intake and satiety. POMC neurons in the arcuate nucleus are activated by leptin and insulin and produce α-MSH, which then activates the MC4R receptor in the paraventricular nucleus, resulting in a satiety signal. The downstream roles of SIM1, BDNF, and TKRB are currently being explored. In contrast, a separate group of neurons expressing NPY and AGRP produce molecules that act as potent inhibitors of MC4R signaling. A dysfunction in these pathways will disrupt energy homeostasis. AGRP, agouti-related protein; α-MSH, alpha melanocyte stimulating hormone receptor; BDNF, brain-derived neurotropic factor; GHR, ghrelin receptor; ISR, insulin receptor; LepR, leptin receptor; NPY, neuropeptide Y; PC1 and 2, proconvertase 1 and 2; POMC, proopiomelanocortin; SIM1*,* single-minded homolog 1 *(Drosophila)*; TRKB, tyrosine kinase receptor.

Indeed, the targeted genetic characterization of naturally occurring obese models, such as the *ob/ob, db/db, fat,* and *tubby* mice, led to the discovery of recessive mutations in the genes encoding leptin (Lep or ob), leptin receptor (Lepr or db), carboxypeptidase E (Cpe or fat), and tubby (Tub) [11]*.* The transfer of this knowledge to clinical cases in which the gene underlying the phenotype was successfully hypothesized validates the roles of some of the aforementioned genes in human monogenic obesity, and clearly positions the leptin/melanocortin pathway as critical in the regulation of whole-body energy homeostasis **(** Figure 1) [12]. In brief, this hypothalamic pathway is activated following the systemic release of the adipokine LEP and its subsequent interaction with the receptor LEPR located on the surface of neurons of the arcuate nucleus of the hypothalamus. The downstream signals that regulate satiety and energy homeostasis are then propagated via proopiomelanocortin (POMC), cocaine- and amphetamine-related transcript (CART), and the melanocortin system [13,14]. While POMC/CART neurons synthesize the anorectic peptide alpha melanocyte stimulating hormone (α-MSH), a separate group of neurons express the orexigenic neuropeptide Y (NPY) and the agouti related protein (AGRP), which acts as a potent inhibitor of melanocortin 3 receptor (MC3R) and melanocortin 4 receptor (MC4R).

Since naturally occurring mutations and the targeted disruption of genes in mouse models *(Lep, Lepr, Pomc, Mc4r,* and *Mc3r)* were all found to have key roles in the same molecular pathway, genes in and associated with the leptin/melanocortin pathway became logical candidates to consider in clinical cases where obesity remained unexplained. Stemming from this hypothesis, several additional genes were found to cause monogenic obesity (Figure 1). First, single-minded homolog 1 *(Drosophila) (SIM1)* was identified in a girl with early-onset obesity and a de novo chromosomal translocation [15]. SIM1 is expressed in the paraventricular nucleus of the hypothalamus, has a role in the melanocortin signaling pathway, and appears to regulate feeding rather than energy expenditure [16]. Second, decreased expression of brain-derived neurotropic factor (BDNF) was recently found to regulate eating behavior [17]. BDNF and its associated neurotrophic tyrosine kinase receptor (TRKB*/*NTRK2) are both expressed in the ventromedial hypothalamus and are proposed to have a role downstream of MC4R signaling [18]. A mutation in TRKB was identified in an 8-y-old male with a complex development syndrome and severe obesity, and further functional in vitro studies have suggested that mutations impair hypothalamic signaling processes [19,20].

A frequent autosomal-dominant form of obesity stemming from mutations in *MC4R* was simultaneously reported by two groups, first in family studies and then in case-control studies [21–24]. Since these initial reports, *MC4R*-linked obesity remains the most prevalent form of monogenic obesity identified to date, representing approximately 2%–3% of childhood and adult obesity [7,25–27]. Mutations have also been reported in the general population, but to a significantly lesser degree [25]. Furthermore, instances have been reported in which individuals in the general population have these mutations but are not obese, suggesting a variable phenotype [25]. Investigating the molecular mechanisms by which loss-of-function mutations in *MC4R* cause obesity has led to a panel of functional anomalies: abnormal MC4R membrane expression, a defect in the agonist response, and a disruption in the intracellular transport of this protein [28]. In contrast with rare monogenic obesities, even careful clinical analysis does not easily detect obesity stemming from *MC4R* mutations because of the lack of additional obvious phenotypes. Thus, the question remains whether there are other forms of obesity with a marked genetic influence, such as that noted for *MC4R* mutation–linked obesity. The recent discovery of rare functional mutations in regions of *POMC* encoding for *α-MSH* and leading to childhood obesity with no other observed anomalies (in contrast to the *POMC* mutations previously described [29]) provides support for the use of genetic screens to identify factors upstream and downstream of MC4R in early-onset and severe human obesity [30].

As the principle goal is to ameliorate the corpulence and metabolic status of obese individuals, cases of monogenic obesity in which gene function is well-characterized can benefit from therapeutic intervention. Leptin therapy in children deficient in this adipokine dramatically reduced body weight, and was accompanied, in some instances, by improved insulinemia and pubertal development [31]. While treatments are not yet available for cases of *POMC-, PC1-, SIM1-, TRKB-,* and *MC4R*-linked obesities, preliminary studies hint that targeted therapies might not be far away.

Recent studies using neuron-specific *LEPR* transgenes, a *POMC* gene-delivery system, and a melanocortin-receptor agonist (MT-II) have all proved efficient in reducing food intake, body weight, and insulin resistance [32–35]. Current MC4R therapeutic peptides are of interest, but have been found to be anorexigenic and to stimulate erectile activity [36–38]; therefore, the development of therapies targeting monogenic obesity will need to circumvent such undesirable side effects. Nevertheless, these studies suggest promising futures for individuals with mutations in genes of the leptin/melanocortin pathway but, as discussed in the remainder of the manuscript, reinforce the enormous challenge lying ahead if therapies are to be developed for individuals with syndromic or polygenic obesity.

## Syndromic Obesity

There are between 20 and 30 Mendelian disorders in which patients are clinically obese, yet are additionally distinguished by mental retardation, dysmorphic features, and organ-specific developmental abnormalities [7,8]. Such cases are referred to as syndromic obesity. These syndromes arise from discrete genetic defects or chromosomal abnormalities, and can be either autosomal or X-linked disorders. However, inasmuch as these cases are well-defined in a clinical context, analyzing the genetic component of these conditions suggests that multiple genes within a biological pathway may produce identical phenotypes [39]. The most common disorders known are Prader-Willi syndrome (PWS), Bardet-Biedl syndrome (BBS), and Alström syndrome, but many others have been reported elsewhere [39].

The most frequent of these syndromes (1 in 25,000 births) is PWS, which is characterized by obesity, hyperphagia, diminished fetal activity, mental retardation, and hypogonadism. This disorder is caused by an absence in the paternal segment 15q11.2–q12 through chromosomal loss. Several candidate genes in the 15q11–13 region of PWS patients have been studied; however, the molecular basis of hyperphagia remains undefined in part because of the fact that none of the currently available PWS mouse models have an obese phenotype [40]. One candidate protein that may mediate the severe hyperphagia of PWS is the gastric hormone ghrelin [41], via its regulation of hunger and stimulation of growth hormone [8]. Ghrelin's role is further implied by the positive findings that growth hormone supplementation is capable of reversing several dysfunctional processes associated with PWS [42,43]; however, in the absence of a suitable experimental model, identifying the genetic components of this syndrome will be challenging.

BBS is characterized by early-onset obesity and rod-cone dystrophy, morphological finger abnormalities, learning difficulties, and renal disease, among other clinical traits. Although originally classified in the clinic as a homogeneous syndrome, BBS has since been associated with at least 11 different chromosomal locations, with several mutations identified within some of the following locations: *BBS1* on 11q13, *BBS2* on 16q21, *BBS3* on 3p13, *BBS4* on 15q22.3, *BBS5* on 2q31, *BBS6* on 20p12, *BBS7* on 4q27, *BBS8* on 14q32.11, *BBS9* on 7p14, *BBS10* on 12q21.2, and *BBS11* on 9q33.1 [44–47]. While BBS was considered to be autosomal-recessive, it has recently been found that the clinical symptoms of certain forms of BBS are related to recessive mutations at one of the *BBS* loci associated with a heterozygous mutation at a second locus, prompting, for the first time, the hypothesis of a triallelic mode of transmission [44,48]. Six genes are characterized in BBS, although their functions remain enigmatic to various degrees. For the *BBS6* locus, positional cloning identified the *MKKS* gene, which codes for a chaperone protein. Mutations identified in *MKKS* result in a shortened chaperone protein and are present in 5%–7% of BBS cases; however, the links between MKKS, its eventual target proteins, and the BBS clinical traits are largely unknown. A newly identified locus, *BBS10,* has recently been found to code for C12orf58, a vertebrate-specific chaperone-like protein, and was found to be mutated in 20% of the populations examined from various ethnic backgrounds [45]. Unlike *BBS6* and *BBS10,* the genes associated with *BBS1, BBS2,* and *BBS4* are very different from *MKKS* and *C12orf58* genes, but it is conceivable that they code for protein substrates of these chaperones [49]. Recently, the gene encoding the E3 ubiquitin ligase TRIM32 was identified as the 11th locus associated with BBS, suggesting that the list of genetic components for this syndrome may yet remain incomplete [47]. Fascinating functional studies performed in single-cell organisms have shown that certain BBS genes are specific to ciliated cells [50]. Ciliated cells have a role in mammalian development, contributing to right/left asymmetry, thus enabling the organs (e.g., heart, liver, and lungs) to be correctly positioned within the biological system. Dysfunction in processes affecting ciliated cells may contribute to the alterations in pigmentary epithelia and structural anomalies noted in certain organs in patients with BBS; however, the relationship between cilia and obesity remains enigmatic [51]. Preliminary findings suggest that cilia formation is not dependent on *BBS* gene functionality [52]; rather, the *BBS* genes may play an important role in intracellular signaling [51]. A recent article by Mak and colleagues used Caenorhabditis elegans to demonstrate the regulation of fat storage by orthologs of both neuronal *TUB* and *BBS1* genes, suggesting the existence of a currently unidentified intertissue signaling pathway that may link ciliated neurons and adipose cells [53]. As such, new fields of research have been opened by the molecular investigation of BBS, most notably regarding the role of ciliated cells in controlling some mechanisms of body-weight regulation.

While syndromic obesity was previously presumed to be under the control of a single gene (and thus was considered monogenic obesity), progress in the post-genomic era has clearly distinguished this type of obesity; however, defining the contribution of multiple genetic factors in a syndrome is significantly more challenging than localizing the single gene involved in monogenic diseases. The difficulty of this task is further amplified if several tissues coordinately regulate phenotype (as possibly exemplified by ghrelin signaling in PWS), indicating that the integrative field of systems biology may hold the key to identifying both the within- and the between-tissue regulators underlying syndromes.

## Polygenic Obesity

**Figure 2:**
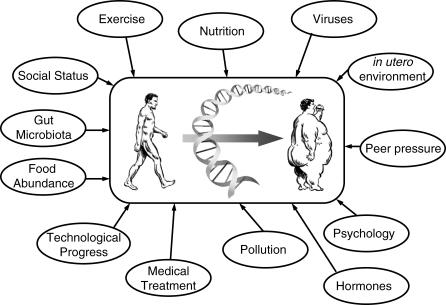
Gene–Environment Interactions in Common Obesity The complex interactions underlying polygenic obesity demonstrate that genetic, social, behavioral, and environmental factors are all capable of influencing the obese phenotype. The DNA strand should be interpreted as taking into account both genetic polymorphisms and the conformation of DNA structure (i.e., degree of methylation influenced by epigenetic events).

Polygenic, or common, obesity arises when an individual's genetic makeup is susceptible to an environment that promotes energy consumption over energy expenditure. Most Westernized societies have an environment that favors weight gain rather than loss because of food abundance and lack of physical activity, thus positioning common obesity as a major epidemic currently challenging these societies. Many excellent reviews have been published in which the genetic complexity and the challenges in dissecting the perturbed biology underlying common obesity have been outlined [7,14,54–56]. Perhaps the greatest obstacle hindering progress is the issue of replication. While independent replication of a novel association is mandatory, it is important to stress that our current degree of understanding of G×E interactions should prevent an unreplicated result from immediately being discarded. Complex traits are highly dependent on G×E interactions; however, inasmuch as this is widely accepted, a question persists: how can one account for and control all possible influences within an experimental design that may affect data interpretation and ultimately experimental conclusions? Although accomplishing this goal is feasible with genetically identical mice, in humans it is nearly impossible. The individual complexity in humans relates to the alleles that influence common diseases in different genetic backgrounds, and to variable genetic combinations eventually influenced by different epigenetic (including in utero) or environmental factors during an individual's lifetime (the role of epigenetics in obesity and its downstream consequences is a field unto itself, and has been thoroughly reviewed in [57,58]) (Figure 2). The precise degree to which genes eventually contribute to complex traits remains poorly defined, and the importance of subtle environmental factors may simply not be appreciated. Thus, as illustrated below by two independent examples, both replicated and unreplicated findings should be considered to be of interest at this early stage in unraveling the genetics of obesity.

**Figure 3:**
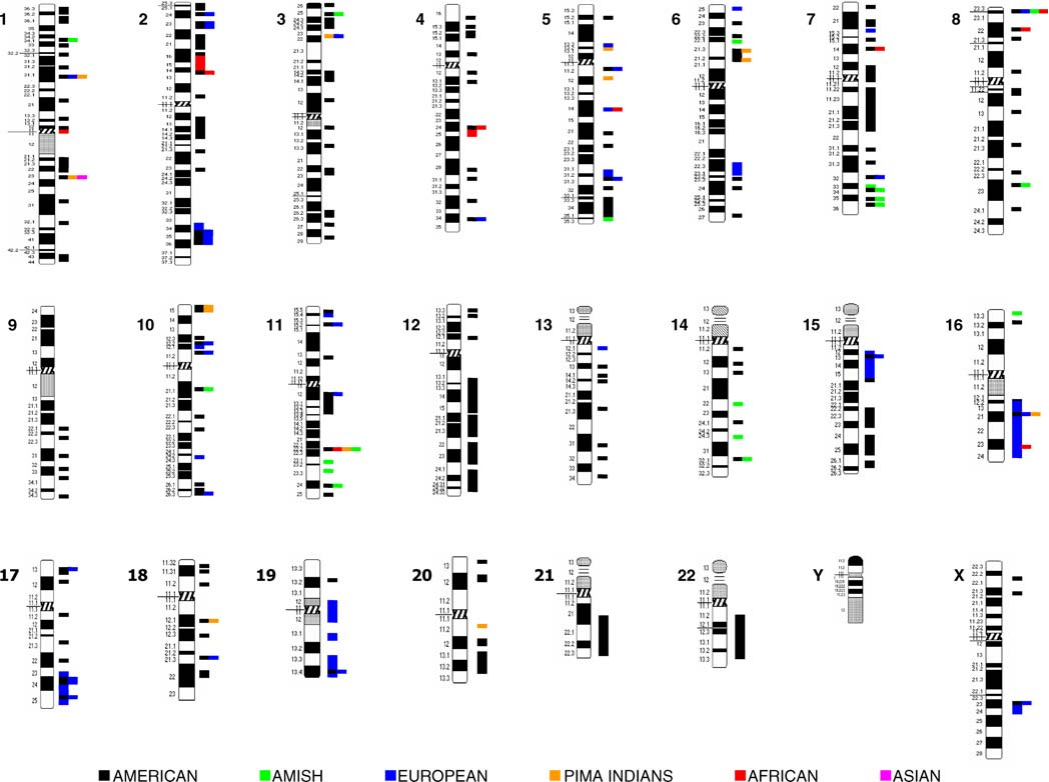
Linkage Studies with Obesity-Related Phenotypes in Six Different Populations All chromosomes but the Y chromosome have been found linked with an obesity-related phenotype (e.g., body mass index, fat mass, waist circumference, and blood pressure) in a least one population. Links for relatively few regions have been replicated in more than one population, as illustrated by colored boxes stacked horizontally. The figure was created using information in the most recent Human Obesity Gene Map update [6]. The American population comprises Caucasian, Hispanic, African, and Asian Americans.

Studies of polygenic obesity are based on the analysis of single nucleotide polymorphisms (SNPs) or repetition of bases (polyCAs or microsatellites) located within or near a candidate gene, where a candidate gene is one that meets a number of criteria, such as its proximity to a quantitative trait locus or its having a phenotypic effect following genetic manipulation (e.g., knock-out and knock-in models) [59]. Should a candidate-gene variant appear promising based on results derived from in vitro and animal-model studies, its association with the obese phenotype is then examined in case-control and family studies [60]; however, unlike monogenic obesity, many genes and chromosomal regions contribute to defining the common obese phenotype (Figure 3) [6,61]. These genes have been implicated in a wide variety of biological functions, such as the regulation of food intake, energy expenditure, lipid and glucose metabolism, and adipose tissue development; however, despite having this ever-increasing gene catalog at our disposal, using this knowledge to unravel the molecular mechanisms underlying obesity is challenging. Indeed, not only is the number of genes associated with obesity high, but variants in some of these genes are demonstrating the importance of polymorphisms in the “interpretation” of environmental stimuli [3]. In contrast to genetically identical mice whose environments can be rigorously controlled, the genetic and environmental diversity in humans has proved problematic for data replication (i.e., to date, only 22 of 244 candidate genes for obesity are supported by at least five positive studies) [6,60,62]. The result can be conflicting findings that cast doubt on potentially interesting candidate genes. For example, an association between three SNPs of *GAD2*, which codes for the 65-kDa subunit of the glutamic acid decarboxylase enzyme, and morbid obesity was identified in a French population following a genome-wide scan [63]; however, independent replication in a larger German population could not be achieved [64]. Although this example raises questions concerning the role of *GAD2* in obesity, it would be premature to discount *GAD2*'s involvement entirely [65]. This notion may also apply to additional candidates (e.g., ectonucleotide pyrophosphatase/phosphodiesterase 1 *[ENPP1]* and solute carrier family 6, member 14 *[SLC6A14]*) emerging from genome-wide scans, as they may also face replication issues.

In many cases, a failure to replicate data is related to the cohort size in which the association was first detected. Indeed, because the contribution of any given gene to the phenotype of a complex trait is often minimal, a large cohort size is required if statistical significance is to be achieved; however, the caveat is that the more associations examined, the greater the risk of type-I errors (i.e., false-positive rate). Statistical approaches such as linkage of disequilibrium threshold values and permutation analyses have proven useful [66], but an ingenious approach recently described by Herbert and colleagues will undoubtedly provide a template for future association studies [67]. Using a multi-stage design, in which the number of SNPs considered is reduced at each step without sacrificing genome-wide significance, the authors selected the top ten SNPs for further analysis, and only one, a SNP variant near the *INSIG2* gene, was associated with obesity. While more common statistical tests, such as the Bonferroni and Hochberg corrections, did not identify this variant, the multi-stage approach employed by the authors proved accurate, as this variant was replicated in four out of five independent populations (no significant association was found using the Nurses Health Study cohort) [60,67]. Thus, the use of novel and creative approaches may provide the means to circumvent classical statistical obstacles in identifying new candidate genes and possible G×E interactions.

## Future Challenges

Our ability to identify promising candidates has progressed rapidly since entering the post-genomic era. Not only is this era characterized by immense progress in molecular biology tools (e.g., microarrays, mass spectrometry, and bioinformatics), it has also prompted the creation of international consortiums that work together towards a common goal [68,69]. Whether consortiums aim to sequence genomes, develop classification terminologies, create publicly accessible databases (such as HapMap), or provide scientific and ethical guidelines for emerging fields, the result is the same: pooling together resources and knowledge from laboratories around the world realizes ambitious goals far more quickly and accurately than an individual research group working alone. For example, well-controlled dietary-intervention studies are currently underway in large populations in Europe, such as in the NUGENOB (http://www.nugenob.com) and Diogenes (http://www.diogenes-eu.org) programs. Such programs, comprising both academic and industrial partners, aim to study G×E interactions and identify those genetic determinants susceptible to environmental stimuli that are capable of influencing obesity development [69,70].

Within such programs, the use of comprehensive platforms (i.e., genetics, transcriptomics, peptidomics, and metabolomics) coupled with clinical data will play a predominant role in elucidating the perturbed functions leading to obesity. Such a “hypothesis-generating” approach promises to identify candidate genes that will undoubtedly implicate novel biological pathways capable of affecting energy status and will enhance our understanding of the evolutionary stages in cellular adaptation during the development of obesity; however, it is important to stress that the association of a gene with a complex trait indicates only a possible risk rather than the causative gene. As such, the research community must err on the side of caution when positioning genes/SNPs in relation to disease. No single SNP will cause obesity; however, a combination of variants exposed to so-called obesogenic environmental stimuli will increase the relative risk that an individual will develop the disease. Alternatively, SNPs may also have a beneficial role by offering a degree of protection against obesity. Recent evidence has demonstrated that the infrequent V103I polymorphism in MC4R is negatively associated with serum triglyceride levels, body mass index, and obesity [71,72]. As such, genetic variations in *MC4R* can lead to both loss and gain of function [73]. Such a finding highlights the importance of searching for SNPs overrepresented in both obese and lean populations. Thus, when looking at the future promise of personalized medicine and nutrition, one must consider both sides of the coin. One side is purely beneficial, where the health and well-being of an individual is improved using information in their genetic blueprint. The other side concerns the ethics of possessing such knowledge, where the details of an individual's genetic makeup may be used inappropriately (e.g., discrimination in society). While new technologies and international consortiums will enable the research community to unravel the genetics of complex traits and realize the ambitious goal of personalization more quickly, the future ethical handling of this information will prove to be a far greater challenge, with the potential for drastic implications.

## Conclusions

In the context of gene–gene and G×E interactions, providing a list of fail-proof guidelines that guarantee the identification of novel candidate genes important in defining complex traits is challenging, as issues such as replication, multiple testing, and sample size will continue to be handled on an experiment-by-experiment basis. Progress in the knowledge of the human genome, the development of comprehensive technologies, and new analytical strategies will permit both the genetic and environmental aspects of complex traits to be addressed simultaneously; however, success will ultimately lie with international consortiums that pool together expertise and resources to define and functionally annotate the genetic factors underlying the various forms of obesity.

## Supporting Information

### Accession Numbers

The UniGene (http://www.ncbi.nlm.nih.gov/entrez/query.fcgi?db=unigene) accession numbers for the genes discussed in the text are as follows: AGRP, Hs.104633; BBS1, Hs.502915; BBS10, Hs.96322; BBS11, Hs.591910; BBS2, Hs.333738; BBS3, Hs.373801; BBS4, Hs.208681; BBS5, Hs.233398; BBS6, Hs.472119; BBS7, Hs.591694; BBS8, Hs.303055; BBS9, Hs.372360; BDNF, Hs.502182; CART, Hs.1707; Cpe, Mm.31395; ENPP1, Hs.527295; GAD2, Hs.231829; INSIG2, Hs.7089; LEP, Hs.194236; Lep, Mm.277072; LEPR Hs.23581; Lepr, Mm.259282; MC3R, Hs.248018; MC4R, Hs.532833; NPY, Hs.1832; NTRK2 (TRKB), Hs.494312; POMC, Hs.1897; SIM1, Hs.520293; SLC6A14, Hs.522109; and Tub, Mm.241469.

## Abbreviations

BBS: Bardet-Biedl syndrome
G×E: interactions between genetic makeup and environment
PWS: Prader-Willi syndrome
SNP: single nucleotide polymorphism
